# Echocardiographic evaluation of the right heart

**DOI:** 10.1007/s00508-018-1330-3

**Published:** 2018-03-19

**Authors:** Matthias Schneider, Thomas Binder

**Affiliations:** Department of Internal Medicine II, Medical University of Vienna, Waehringer Guertel 18–20, 1090 Vienna, Austria

**Keywords:** Right ventricular function, Transthoracic echocardiography, TAPSE, Longitudinal strain, 3D echocardiography

## Abstract

Symptoms of right ventricular failure include dyspnea, a reduction in exercise capacity, and fluid retention. Right ventricular (dys)function strongly influences functional state and survival. The right ventricle is directly involved in a variety of diseases. A thorough analysis of right ventricular size and function, as well as estimation of pulmonary artery pressures is an important part of every echocardiographic examination. This review analyses the most commonly used parameters for quantification of right ventricular function. It gives a practical approach for estimation of right ventricular size and function, as well as pulmonary artery pressure.

## Echocardiographic evaluation of the right heart

The right ventricle is directly involved in a variety of diseases. Right ventricular size and function play a crucial role in patients with pulmonary hypertension, cardiomyopathies, pulmonary embolism, right ventricular infarction, pulmonary and tricuspid valvular heart disease and intracardiac shunts. In recent years, right ventricular function in left heart disease has received more and more attention [6, 16, 20]. Symptoms of right ventricular failure include dyspnea, a reduction in exercise capacity, and fluid retention [33]. Right ventricular (dys)function strongly influences functional state and survival [30].

## Right ventricular anatomy, physiology, and disease

The shape of the right ventricle (RV) has been compared to that of a bagpipe and consists of an inflow and an outflow compartment. The RV and the left ventricle (LV) are closely linked together. The interventricular septum connects the two ventricles. They share the same pericardial space and have mutual epicardial fibers. The RV generates the same stroke volume as the LV but due to low resistance in the pulmonary vasculature it requires only one quarter of the stroke work. This explains the thin right ventricular wall as compared to the left ventricular myocardium [32]. When pulmonary pressures rise and/or right ventricular volume overload occurs, the RV reacts with hypertrophy, dilatation, and increased contractility.

The gold standard for quantification of size, ejection fraction, and stroke volume of the RV is cardiac magnetic resonance imaging (MRI). The gold standard for the measurement of pulmonary pressures is right heart catheterization; however, echocardiography is usually used as the first diagnostic tool to assess size and function of the right heart. As an echocardiographer, it is important to always keep in mind the power but also the pitfalls of echocardiographic assessment of pulmonary pressures and right ventricular size and function. Lang et al. published the most recent recommendations for cardiac chamber quantification by echocardiography in adults in 2015 [18], amending the guidelines for the echocardiographic assessment of the right heart in adults by Rudski et al. [25]. Both manuscripts are the basis of the following review.

## Right ventricular size

Assessment of the size of the RV in 2D echocardiography is challenging. Common diseases that lead to right ventricular dilatation, such as left-to-right shunts, pulmonary valve disease, tricuspid regurgitation, pulmonary hypertension, right ventricular infarct and cardiomyopathies, e.g. arrhythmogenic right ventricular dysplasia (ARVD) have to be excluded. In patients with chronic pressure and/or volume overload, follow-up examinations have to clarify if the patients are stable or if there is deterioration in size and/or function. Precautious analysis of the size of the RV has to be performed and the examiner must ensure that measurements are made in a comparable way to the previous studies. The right heart has to be imaged from multiple acoustic windows, and the report should be based on a comprehensive interpretation of all findings.

Echocardiography allows reliable detection of a severely or moderately dilated RV. While the ratio between the size of the LV and the RV is more than 1.3 under physiological circumstances, the RV often exceeds the size of the LV in patients with moderate or severe right ventricular dilatation (ratio <1). It is more difficult to differentiate “normal” from “abnormal” when the RV is only mildly dilated. In such situations, quantitative parameters become very important.

**Fig. 1:**
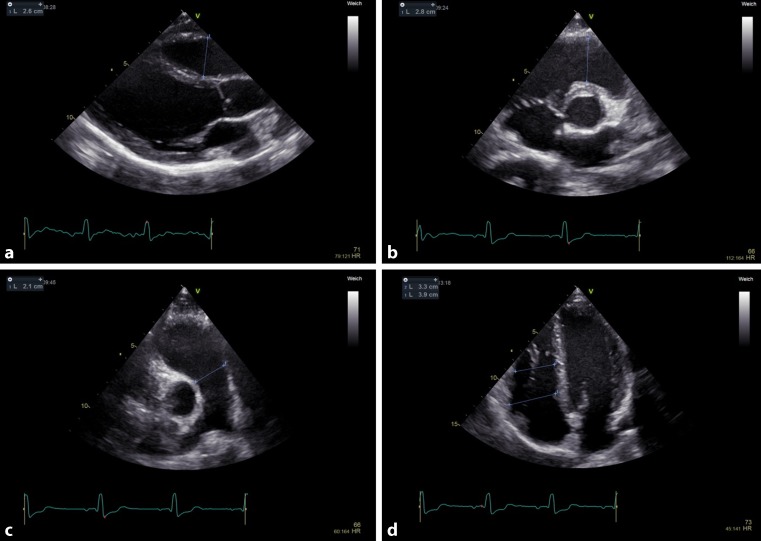
Systematic evaluation of right ventricular size from four standardized transthoracic views and their normal values. **a** Proximal outflow tract parasternal long axis view; >30 mm abnormal, normal range 20–30 mm. **b** Proximal outflow tract parasternal short axis view; >35 mm abnormal, normal range 21–35 mm. **c** Distal outflow tract; >27 mm abnormal, normal range 17–27 mm. **d** Apical right ventricle at base; abnormal: >41 mm, normal range 25–41 mm. Apical right ventricle at mid-level; abnormal: >35 mm, normal range 19–35

The normal RV has a triangular shape in the 4‑chamber view. Transverse measurements differ significantly the closer one gets from the base (tricuspid annulus) to the apex. In the RV focused 4‑chamber view three measurements can be performed. The RV at its base, at mid-level and in the longitudinal dimension. The second imaging window is the RV outflow tract. First the proximal diameter should be measured in the parasternal short axis view and/or in the parasternal long axis view. Finally, the distal diameter of the RV outflow tract should be measured at the level of the pulmonary valve insertion [18]. Normal values and cut-offs for abnormal measurements are shown in Fig. 1.

In contrast to the LV, no exact geometric model exists for the RV, which would allow for volumetric assumptions by 2D echocardiography. The size of the RV is underestimated in 2D echocardiography as opposed to cardiac MRI. This especially applies to patients with congenital heart disease and to patients with RV volume overload [17]. The use of 3‑dimensional (3D) echocardiography has received wide attention due to the possibility to measure true 3D volumes of the RV chamber instead of 2D diameters at different levels [18, 28]; however, the method is rarely applied in daily clinical practice. If RV hypertrophy is suspected the RV wall thickness can be measured. Right ventricular hypertrophy is present if the free lateral wall of the RV exceeds 5 mm. The recommended view for this measurement is the subcostal 4‑chamber view.

## Right ventricular function

The main role of the RV is to sustain an effective cardiac output. Stroke volume of the RV is predominantly generated by longitudinal shortening rather than by reduction of the cavity diameter (radial function) as is the case in the LV [24]. Due to the complex anatomy of the RV, echocardiographic evaluation of RV function is often difficult.

**Fig. 2:**
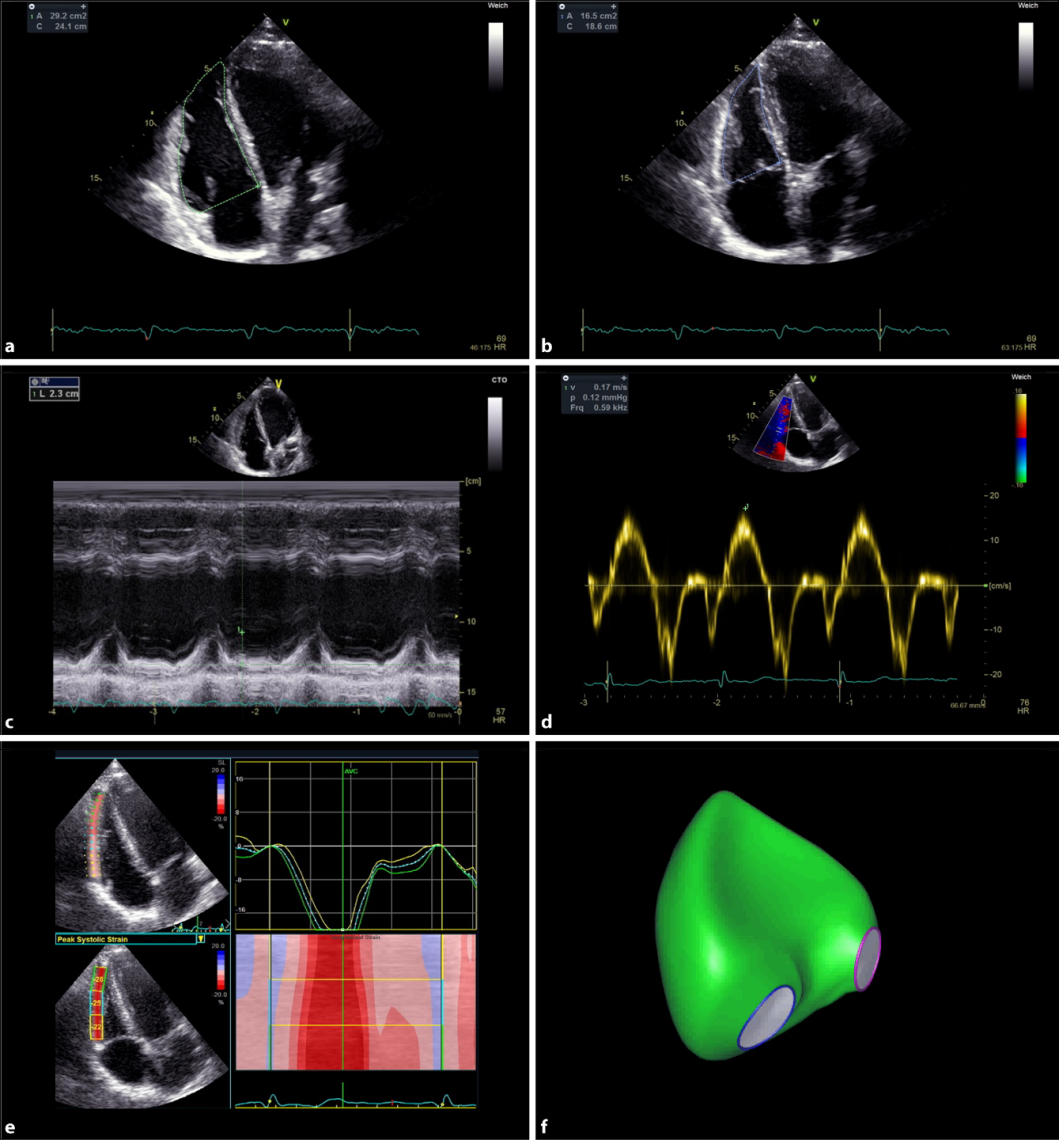
Evaluation of right ventricular function: RV end-diastolic (panel **a**) and end-systolic (panel **b**) area for calculation of right ventricular fractional area change. Tricuspid annular plane systolic excursion (TAPSE) (panel **c**) and Tissue Doppler of the free lateral wall (S’) (panel **d**), longitudinal strain of the free lateral wall of the right ventricle (panel **e**), and transthoracic 3D reconstruction of the right ventricle (panel **f**)

Visual examination is the most commonly used method to quantify right ventricular function (RVF). There are many pitfalls that can lead to overestimation or underestimation of function. If used as a single parameter, visual examination proved to be an inaccurate method for evaluation of RVF [4, 19]; therefore, the guidelines suggest using at least one other parameter to quantify RVF. Several echocardiographic RVF parameters have been established and validated. Each of the parameters has significant limitations and pitfalls. In a recent survey our group investigated which methods are applied in daily clinical practice for the assessment of RVF. Of the participants from Austria and Germany, 80% used visual examination, 72% used TAPSE, 19% used S’, and 4% used fractional area change. The remaining methods are only rarely applied (unpublished data). This review therefore concentrates on these regularly applied methods. In addition, the new and promising parameters global longitudinal strain and 3D echocardiography are discussed (Fig. 2).

### Tricuspid annular plane systolic excursion (TAPSE)

TAPSE is the most commonly used parameter for RVF quantification. In an apical 4‑chamber view the maximum systolic excursion of the lateral tricuspid annulus is measured by M‑mode. The tracing can be acquired quickly and its interpretation is straightforward. A low TAPSE value is associated with lower cardiac index and worse survival [8, 11]. TAPSE is based on a one-dimensional measurement and is therefore only partially representative of global RV function. A TAPSE of <17 mm indicates RV dysfunction [18].

### Tissue Doppler of the free lateral wall (S’)

The Tissue Doppler of the free lateral wall (S’) measures the longitudinal velocity (base to apex) of the tricuspid annular plane by tissue Doppler imaging. The sample volume is placed in the basal segment of the free lateral wall of the right ventricle or into the tricuspid annulus. Since the method is angle-dependent, the Doppler beam has to be aligned parallel to the motion of the free lateral wall towards the apex. The measurement is reproducible and easily obtained. A comparison with MRI-derived RV ejection fraction (RVEF) showed a significant correlation with S’ [22]. An S’ value <0.095 m/s indicates RV dysfunction [18].

### Fractional area change (FAC)

The fractional area change (FAC) is a 2D surrogate for RVEF. It is performed by tracing the endocardial RV borders during diastole and systole and is calculated by the following formula: 100 × (RV-Area end-diastolic [ED] − RV-Area end-systolic [ES]) / RV-Area ED. A RV focused 4‑chamber-view is essential to ensure that the entire endocardial border is visible in end-systole and end-diastole. Trabeculations and the tricuspid valve leaflets are included when tracing the area. The FAC showed good correlation with MRI-derived RVEF [1]. It is an independent predictor of morbidity and mortality in patients after myocardial infarction [2], in patients undergoing cardiac surgery [23] and in patients with pulmonary hypertension [3]. A value of less than 35% indicates RV systolic dysfunction [18].

### Longitudinal strain

The longitudinal strain is the percentage of systolic shortening of the myocardium. One can assess both regional and global contractile function. Global longitudinal strain is a well-established tool to detect early LV dysfunction but it can also be used for quantification of RVF. Global strain of the free lateral wall of the RV is calculated by averaging peak systolic strain of the three segments of the free lateral wall in an RV focused 4‑chamber-view. Higher negative strain values represent better systolic function. Numerous studies showed that RV strain is a powerful method to assess RVF. It is a predictor of survival in patients with pulmonary hypertension [14] and correlates well with RV stroke volume index, right atrial pressure [26], and RVEF [7]. A free-wall strain of less than −20% (less meaning smaller absolute number) indicates reduced RVF. Mean values for RV strain in healthy controls are −29% ± 4.5 [18].

### 3D measurements of RV function and size

The prognostic value of RVEF was shown in several studies. Radionuclide angiography derived RVEF ≥35% vs. 25–35% vs <25% predicted survival as 93% vs 77% vs 59%, respectively, in patients with left heart failure [13]. Loss of RVF measured my MRI derived RVEF is associated with a poor outcome in pulmonary arterial hypertension (PAH) patients [31]. In an MRI study, van Wolferen et al. could show that RV dilatation in addition to low stroke volume and impaired LV filling independently predict mortality. A further decrease in stroke volume, an increase in RV dilatation and a further decrease of LV end-diastolic volume at 1 year follow-up were the strongest predictors of mortality [34].

So far, the problem for echocardiographers was the unreliable estimation of RVEF via 2D echocardiography since it is not able to calculate true volumes. This limitation is overcome with 3D echocardiography, which does not rely on geometric assumptions but calculates true 3D volumes. The RVEF derived by 3D echocardiography proved to be an independent predictor of adverse cardiovascular outcomes in patients with various cardiovascular diseases [21]; however, the 3D method requires special transducers, dedicated hardware and software, and is associated with a higher cost and additional time to perform the measurements. In addition, good image quality is mandatory for correct analysis. The future will show to which degree 3D echocardiography will be implemented into daily clinical practice when evaluating RVF.

Many conditions have been described where TAPSE and S’ do not reflect true RVF. These include patients after cardiac surgery, tetralogy of Fallot, and tricuspid regurgitation [12, 15, 27, 29]. In particular, in these cases new methods such as global longitudinal strain and 3D measurements of RV volumes and RVEF deserve attention for future studies.

### Other parameters

Other parameters such as RV myocardial performance index (RIMP) and the rate of RV pressure rise during early systole (dP/dt) proved to correlate with pulmonary vascular resistance [5] and to predict a reduced RVEF [22]. They are rarely used in daily clinical practice and therefore will not be discussed in this review.

### Not just a conduit: The right atrium

**Fig. 3:**
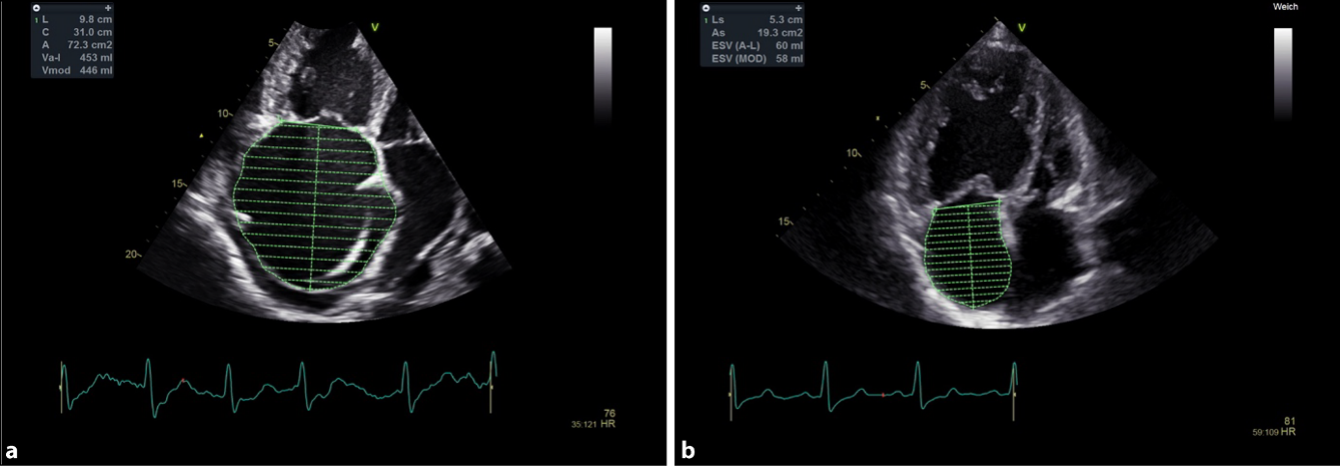
Right atrial size in two patients with pulmonary hypertension. Patient A with decompensated right heart failure (RA volume 453 ml). Patient B with compensated clinical state (RA volume 60 ml)

The right atrium (RA) assists filling of the right ventricle [24]. When the tricuspid valve is closed RA is a reservoir for systemic venous return. When the tricuspid valve is open, the RA acts as a passive conduit in early diastole and as an active pump in late diastole [10, 25]. A dilated RA can be a sign of volume and/or pressure overload. Elevated RA pressure is a sign of poor RVF. Austin et al. could show that elevated estimated RA pressure by echocardiography is a predictor for mortality [3]. Therefore, RA size should be considered as an important puzzle piece in the interpretation of RVF. It is recommended to assess RA volume by using single-plane area-length or disk summation techniques in an apical 4‑chamber view (Fig. 3). Normal values for women are 21 ± 6 ml/m^2^ and for men 25 ± 7 ml/m^2^ [18]. The guidelines for diagnosis and treatment of pulmonary hypertension consider an RA area >18 cm^2^ as an echo-sign for elevated RA pressures [9].

## The role of echocardiography in pulmonary hypertension

Estimating pulmonary artery pressures is an important part of every echocardiographic evaluation of the right heart. Echocardiography plays a crucial role in the diagnosis of pulmonary hypertension. Underestimation of pulmonary pressures can lead to delayed or false diagnosis. Overestimation can result in unnecessary right heart catheterization and possible complications.

**Fig. 4:**
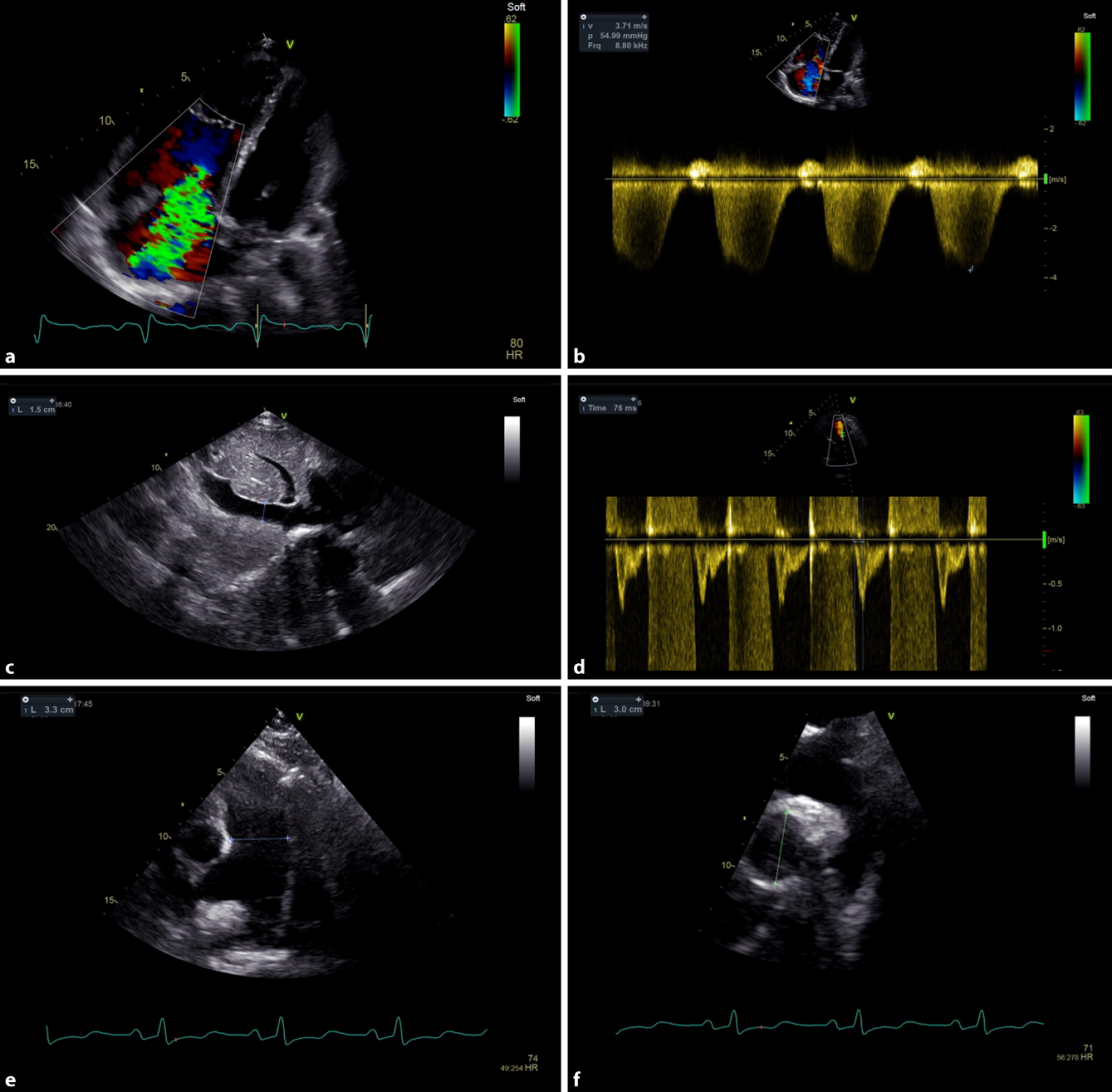
Systematic evaluation of signs of pulmonary hypertension. Maximum jet velocity over tricuspid regurgitation (panels **a** and **b**). Diameter and respiratory change of inferior vena cava (panel **c**). Pulmonary valve acceleration time (panel **d**). Dilatation of the pulmonary artery (panel **e**) showing the pulmonary trunk in the parasternal short axis and panel **f** showing the right pulmonary artery from suprasternal angulation

The guidelines for the diagnosis and treatment of pulmonary hypertension (PH) suggest the following approach for echocardiographic assessment of patients with suspected PH: maximum tricuspid regurgitation (TR) velocity and other echo signs of PH should be evaluated (e. g. dilated RV, flattening of the interventricular septum, short pulmonary valve acceleration time, pulmonary artery [PA] diameter >25 mm, dilated RA, decreased inspiratory collapse of inferior vena cava; Fig. 4).

Calculated systolic pulmonary artery pressure (Bernoulli equation) is not exact and the value should not be used to grade severity of PH. Instead, maximum tricuspid regurgitant jet velocity is used as a surrogate of RV systolic pressure. Due to the angle dependency of the Doppler method, it has to be imaged from different views.

If tricuspid regurgitation velocity is ≤2.8 m/s and in the absence of other echo signs of PH, the probability for PH is low. If TR velocity is ≤2.8 m/s or there is no sufficient signal but there are echo signs of PH or if TR velocity is 2.9–3.4 m/s and there are no echo signs for PH, then the PH probability is intermediate. In the presence of a TR velocity 2.9–3.4 m/s together with other echo signs or if TR velocity is above 3.4 m/s regardless of the presence of other echo signs, the probability for PH is high [9].

## Conclusion

A comprehensive assessment of the RV is an integral part of every echocardiographic examination. Dilatation of the RV must be ruled out using multiple views, visual examination, and measurements of RV size. Ideally, multiple parameters should be used to determine right ventricular systolic function. Validated and commonly used parameters are: visual examination, TAPSE, S’, FAC, longitudinal strain, and 3D echocardiography. The standardized approach suggested by the PH guidelines should be followed when evaluating pulmonary pressures.
